# Effect of Morphology/Structure on the Phase Behavior and Nonlinear Rheological Properties of NR/SBR Blends

**DOI:** 10.3390/gels8070425

**Published:** 2022-07-07

**Authors:** Li Yang, Xuanyu Shi, Shihao Sun, Jun Zhong, Xiaofeng Yu, Danling Wang, Yihu Song, Min Zuo, Qiang Zheng

**Affiliations:** 1 Department of Polymer Science and Engineering, Zhejiang University, Hangzhou 310000, China; 12029060@zju.edu.cn (L.Y.); 12129019@zju.edu.cn (X.S.); 12029051@zju.edu.cn (S.S.); 22029042@zju.edu.cn (J.Z.); 22129036@zju.edu.cn (X.Y.); s_yh0411@zju.edu.cn (Y.S.); 2 Zhongce Rubber Group Co., Ltd., Hangzhou 310000, China; yogidan@163.com

**Keywords:** NR/SBR blend, morphology, nonlinear viscoelasticity

## Abstract

The evolution of the morphology/structure and the nonlinear viscoelasticity of rubber blends under large amounts of strain are key scientific issues for the design and manufacture of rubber blends. The rheological responses of natural rubber/styrene-butadiene rubber (NR/SBR) blends are traced over a wide range of blend compositions to gain an insight into the effect of blend morphology on their nonlinear viscoelasticity. We also prepare NR + SBR physical blends without melt mixing to distinguish the contributions of composition and blend morphology to the viscoelastic response. The microscopic heterogeneous gel-like structure of NR/SBR blends may remarkably weaken their strain softening and improve their modulus hysteretic recovery under large strain, which may be attributed to the heterogeneous microscopic deformation for the NR and SBR phases. Furthermore, additional elastic contribution resulted from the increasing interfacial energy of domain deformation. This may provide some new insights into the effect of blend morphology on the Payne effect of rubber blends.

## 1. Introduction

Blending different rubbers (natural or synthetic) is one of the facile and effective strategies to prepare the rubber products with a desired performance, as adjusting their chemical structure requires a lot of energy and cost to improve their properties. At present, most high-performance rubbers are multiphase/multicomponent systems, the properties of which are mainly determined by their morphology and structure. The resulting morphology can be influenced by many factors, such as blend ratio, blend composition, microstructure, and their viscosity, polarity, and mixing process [1]. Studies have found that in styrene-butadiene rubber (SBR)-based mixtures, increasing the mixing temperature leads to a finer dispersion of cyclic butylene terephthalate (CBT) oligomer compounds, which further improved the mechanical properties [2]. Hence, it is crucial to understand the relationship between their morphology/structure and properties for optimizing the performance of their final products. The molecular miscibility in most polymer blends is impossible because of the difference in surface free energy. When the phase decomposition and the combination processes [3] are in equilibrium state, the equilibrium morphology is achieved. Furthermore, the molecular weight and molecular weight difference between the two phases also affect their morphology. The smaller phases are related to similar molecular weights, and the domain size increases with the increasing molecular weight differences of the components [4]. Viscosity also affects the domain size in the same way, and the greater viscosity difference between components may result in the formation of larger domains [5,6,7,8].

Natural rubber (NR), as a renewable biosynthetic polymer, is the most commonly used general rubber [9,10,11]. Although NR has excellent elasticity, flex resistance, and mechanical strength, its abrasion resistance and wet skid resistance is slightly poorer than other rubbers. On the other hand, SBR has excellent abrasion resistance and wet skid resistance, but its elasticity is slightly poor. Therefore, an NR/SBR blend can combine the advantages of both to improve its wear resistance, oxidative stability, and wet skid resistance and, as such, can have broad application prospects in rubber products, especially in the tire industry [12,13]. The NR/SBR blend is also used in many other aspects, such as sealing elements [14], rubber soles [15], and the surface corrosion protection of metallic tanks [16]. Most investigations are focused on the improvement of their mechanical properties and their extensive application, but there are few in-depth scientific studies on the morphological structure evolution and nonlinear viscoelasticity of NR/SBR blends.

It is well-known that NR is hardly miscible with SBR at the molecular level in most cases, and that the microstructure of SBR (including the vinyl and styrene contents) may affect the morphology of the NR/SBR blend [17,18,19,20]. In previous research, the effect of domain morphology on the physical and dynamic mechanical properties of the NR/SBR or NR/BR blend and their filled compounds has been discussed preliminarily and macroscopically [21,22,23]. For the safe driving of the tire, it is not enough to only pay attention to their mechanical performance, and more attention should also be paid to their nonlinear viscoelastic response and morphology evolution during periodic deformation at large strain amplitude (*γ*). Rubber materials subjected to large-*γ* oscillation usually exhibit a typical nonlinear softening response, namely the Payne effect [24,25,26]. Storage modulus (*G*′) drops with the increasing strain amplitude (*γ*) beyond the linear regime upon loading, and then partially recovers with decreasing *γ* upon unloading, while loss modulus (*G*″) approaches a maximum (weak strain overshoot, WSO) or decreases synchronously with *G*′ [27,28]. A sufficient understanding of the nonlinear viscoelastic response and morphology of rubber blends will facilitate the design and preparation of rubber materials with high performance [29,30,31].

**Figure 1:**
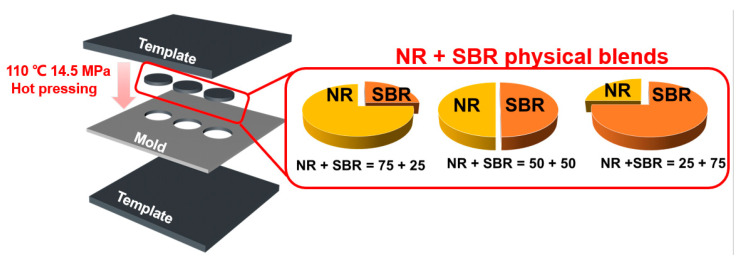
Preparation scheme of NR + SBR physical blends with different weight ratios for rheological measurements. Here, NR and SBR disks with a thickness of 2 mm and a diameter of 25 mm were cut and combined laterally at different weight ratios, and then hot-pressed without internal mixing. The gold and orange parts represent NR and SBR, respectively.

In this work, the rheological responses of NR/SBR blends are traced over a wide range of blend compositions to gain an insight into the effect of continuous or droplet-matrix morphology on their nonlinear viscoelasticity. We select SBR with a styrene content of 40% and a vinyl content of 18% to blend with NR and form the immiscible NR/SBR blends. We compare the rheological behavior of NR/SBR melt blends with that of their NR + SBR physical blends without internal mixing (the preparation scheme as shown in Figure 1) to distinguish the contribution of heterogeneous morphology/structure for NR/SBR blends to the nonlinear viscoelastic response. This allows us to understand the Payne effect mechanism of rubbery blends from another point of view.

## 2. Results and Discussion

### 2.1. Effect of SBR Content on the Glass Transition Temperature (T_g_) and Morphology of NR/SBR Blends

**Figure 2:**
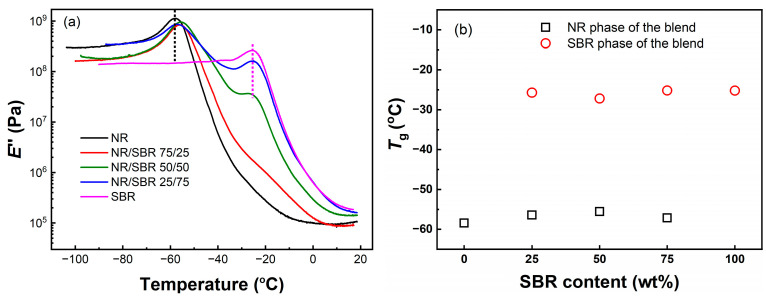
(**a**) Loss modulus *E*″ as a function of temperature for NR/SBR blends. (**b**) Glass transition temperature *T*_g_ as a function of SBR content for NR/SBR blends.

Glass transition temperature (*T*_g_) is essential for tire tread rubber, as it is the factor which largely determines the wear resistance, wet skid resistance, rolling resistance, and low temperature driving performance of the tire. For an immiscible binary blend, two glass transition temperatures different from those of each component in the blend can be observed, and their *T*_g_ variation is related with the compatibility of the blend [17,32]. Here, dynamic mechanical analysis (DMA) was employed to study the SBR content dependence of *T*_g_ for NR/SBR blends in the dual cantilever mode during gel-to-glass-like transition. Figure 2a shows the loss modulus (*E*″) as a function of temperature for NR/SBR blends with different weight ratios, respectively, while Figure 2b shows their *T*_g_ values as a function of SBR content. Here, two obvious *T*_g_ peaks can be observed for the NR/SBR blends with the weight ratios of 50/50 and 25/75, while, for the NR/SBR (75/25) blend, only one remarkable *T*_g_ peak with a shoulder peak can be observed. Furthermore, the *T*_g_ values for the NR and SBR phases in the NR/SBR blends are close to those for pure NR and SBR, indicating that the weak compatibility between NR and SBR may be attributed to the high styrene content (40%) of SBR in our study. On the other hand, the temperature distance between the two glass-to-gel-like transitions in the NR/SBR (50/50) blend is a little narrower than those in NR/SBR (75/25) and (25/75) blends, indicating that the compatibility of NR with SBR may be affected by their blend morphology.

**Figure 3:**
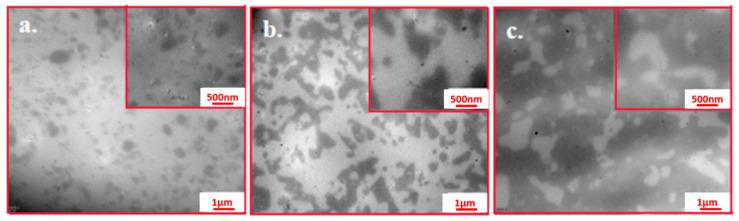
TEM micrographs of (**a**) NR/SBR (75/25), (**b**) NR/SBR (50/50), and (**c**) NR/SBR (25/75) blends.

The morphology of the NR/SBR blends with different weight ratios was observed by transmission electron microscope (TEM, as shown in Figure 3. The dark and bright regions in the TEM images correspond to the SBR and NR domains, respectively. The morphology of the NR/SBR blends may be apparently associated with their blend composition. The NR/SBR (75/25) blend shows the sea-island structure with small SBR droplets in the NR matrix. Moreover, the SBR domains are nearly spherical, with a diameter of smaller than 1 μm, and the domain boundary is more indistinct, resulting in weak glass transition of the SBR phase observed in their DMA curve. The NR/SBR (50/50) blend exhibits a co-continuous morphology, while the NR/SBR (25/75) blends show the coexistence structure of droplet and percolation for NR domains in the SBR matrix, owing to the higher viscosity of the NR phase. Hence, the domain size of irregular droplets in the NR/SBR (25/75) blend is also larger than that of the spherical droplets in the NR/SBR (75/25) blend.

### 2.2. Linear Rheological Behaviors

**Figure 4:**
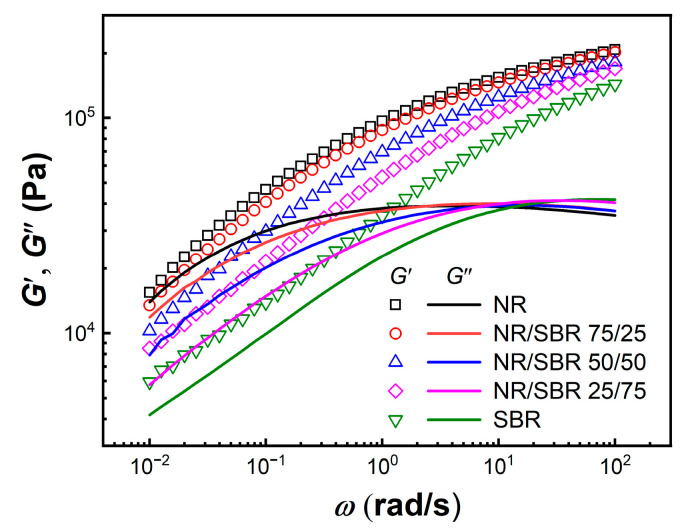
Storage moduli *G*′ (open symbols) and loss moduli *G*″ (solid lines) as a function of frequency *ω* at *γ* = 0.5% and 100 °C for the NR, SBR, and NR/SBR blends.

**Figure 5:**
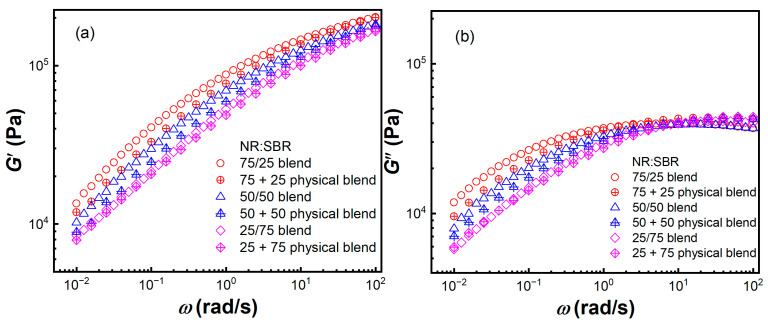
(**a**) Storage moduli *G*′ and (**b**) loss moduli *G*″ as a function of frequency *ω* at *γ* = 0.5% and 100 °C for the NR/SBR blends and NR + SBR physical blends.

The morphology of polymer blends may affect their linear viscoelastic response at the low frequency *ω* region [33]. Small amplitude oscillatory shear (SAOS) tests were carried out at 100 °C (*γ* = 0.5%) for NR/SBR blends within the linear viscoelastic regime before large amplitude oscillatory shear (LAOS) tests. Figure 4 shows dynamic storage moduli *G*′ and loss moduli *G*″ as a function of *ω* for NR/SBR blends with various weight ratios. The NR, SBR, and NR/SBR blends all exhibit nonterminal flow behavior (*G*′ > *G*″) in the experimentally achieved *ω* range at 100 °C. The values of *G*′ and *G*″ of the NR/SBR blends increase with the increasing content of NR within the whole investigated *ω* region. At high *ω*s, the values of *G*′ and *G*″ of NR/SBR lie between the moduli values of NR and SBR. To distinguish the contribution of morphology and components of NR/SBR blends to *G*′ and *G*″ at low *ω*s, three kinds of NR + SBR physical blends without internal mixing are prepared, as shown in Figure 1. Figure 5 shows the *ω* dependence of *G*′ and *G*″ for the NR/SBR blends and NR + SBR physical blends at 100 °C. The dynamic moduli of NR/SBR blends at low *ω*s are obviously higher than those of the NR + SBR physical blends, which may be attributed to the presence of the heterogeneous gel-like structure in NR/SBR blends, as shown in Figure 3. Hence, the enhancement of the dynamic viscoelastic response for NR/SBR blends is attributed to their morphology evolution and interfacial tension between two phases [30]. The modulus deviation of the NR/SBR (25/75) blend is less marked than those of the NR/SBR (75/25) and (50/50) blends, resulting from the low deformability of the dispersed NR phase with high viscosity under small strain. Furthermore, the more remarkable modulus increment of the NR/SBR (50/50) blend may be attributed to the formation of interpenetrating gel-like networks. Here, the effect of morphology (droplet-matrix or co-continuous) for NR/SBR blends on the *G*′ at low *ω*s is similar to that for some other binary blends of poly (methyl methacrylate) (PMMA)/poly (styrene-co-maleic anhydride) (SMA) [34] and fluorescently labeled polystyrene (FLPS)/poly (styrene-co-acrylonitrile) (SAN) [35]. Here, the samples are all in the linear viscoelastic regime, and the linear rheological behaviors of NR + SBR physical blends are just related with the contribution from two components.

### 2.3. Nonlinear Rheological Behavior

**Figure 6:**
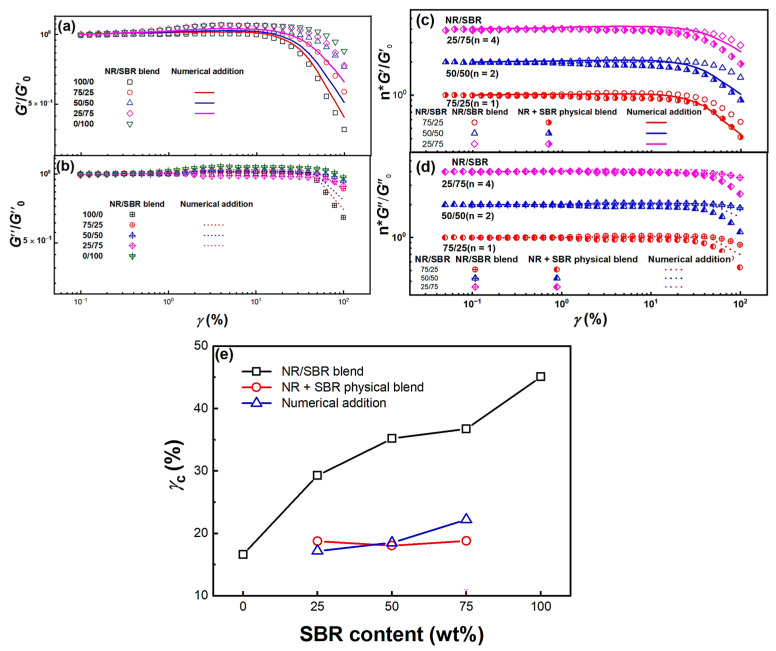
Normalized *G*′/*G*′_0_ and *G*″/*G*″_0_ as a function of *γ* for NR/SBR blends (**a**,**b**) and NR + SBR physical blends (**c**,**d**) at 100 °C and 1 rad/s. The straight and dashed lines are normalized *G*′/*G*′_0_ and *G*″/*G*″_0_ values obtained by linear numerical addition of neat NR and SBR by weight ratio. Here, the normalized moduli for the NR/SBR blends and NR + SBR physical blends with different weight ratios are vertically shifted by a factor of n to clearly show their difference. The corresponding n value is labelled in the bracket behind the NR/SBR ratio. (**e**) Onset strain *γ*_c_ for the Payne effect as a function of SBR content for the NR/SBR blends, NR + SBR physical blends, and modulus numerical addition.

In a large number of studies, the rapid decrease in modulus upon shear at large amplitudes in the nonlinear viscoelastic regime might be attributed to the destruction of the particle–particle network of the filler in the rubber matrix [36,37,38], the destruction of the weak polymer-filler particle network [38,39], the desorption of rubber molecular chains [40], and the destruction of the chemical network [36,41], etc. However, these mechanisms could hardly explain the Payne effect of NR, SBR, and NR/SBR blends. In order to investigate the effect of blend morphology on the strain (*γ*) response of NR/SBR blends, dynamic *γ*-sweeps are performed at 100 °C and *ω* = 1 rad/s. The normalized *G*′/*G*″_0_ and *G*″/*G*″_0_ values for the NR, SBR, and NR/SBR blends as a function of *γ* are shown in Figure 6a,b. Here, *G*′_0_ and *G*″_0_ are the storage and loss moduli at low *γ* in the linear viscoelastic region, respectively. For immiscible blends, their nonlinear viscoelastic response is not only related to the components in the blends, but is also related to the blend morphology. Here, the hollow symbols are the experimental results, and the lines are fitted by linear numerical addition as follows [42]:

*G* *_blend_ = *φ⋅G* *_SBR_ + (1−*φ*)*⋅G* *_NR_ (1)

in which *φ* is the content of SBR.

The linear viscoelastic regime for SBR is remarkably longer than that of NR, and the linear region for NR/SBR blends extends with the increasing SBR content. Furthermore, the fitted numerical addition results may just correspond to the modulus contribution from the NR and SBR components, and the rapid decrease in dynamic moduli for NR/SBR blends from experimental results obviously occurs at the higher *γ*_c_. Compared with their numerical addition results, the extension of the linear viscoelastic regime for the NR/SBR blend may be attributed to the contribution from the heterogeneous structure in NR/SBR blends. Here, we also investigate the difference of dynamic *γ*-response for the NR/SBR blends and NR + SBR physical blends to reveal the effect of blend morphology on their Payne effect. Figure 6c,d show the *γ* dependence of normalized *G*′/*G*′_0_ and *G*″/*G*″_0_ for NR/SBR blends and NR + SBR physical blends, respectively. To show the influence of blend composition and morphology on their Payne effect clearly, their normalized moduli are vertically shifted by a factor of n for the blends with different weight ratios, and the corresponding n values are labelled. For three systems with different compositions, the amplitude of the modulus decay for NR/SBR blends is much weaker than that for their corresponding NR + SBR physical blends. Here, without the incorporation of nanoparticles, the Payne effect for NR/SBR blends and NR + SBR physical blends may mainly originate from the shear-induced overstraining [43], over-orienting [39], and disentanglement activated by the convective constraint release [27,44,45] in the nonlinear viscoelastic region. The weakening of the Payne effect for NR/SBR blends may be related with their interface contribution of heterogeneous structure, especially in the NR/SBR 50/50 blend (co-continuous morphology). Furthermore, the modulus decay amplitude of NR + SBR physical blends is also larger than that of their corresponding numerical addition results. The critical strain for the onset of nonlinearity (*γ*_c_) increases with increasing SBR content, and the *γ*_c_ values for NR/SBR blends are obviously higher than those for the NR + SBR physical blends and numerical addition results (as shown in Figure 6e), indicating that the presence of the heterogeneous structure in NR/SBR blends may extend their linear viscoelastic region and delay the occurrence of rheological nonlinearity. The *γ*_c_ values for the NR + SBR physical blends are very close to that of neat NR and independent of SBR content, indicating that the strain softening of NR + SBR physical blends might be just dominated by NR.

**Figure 7:**
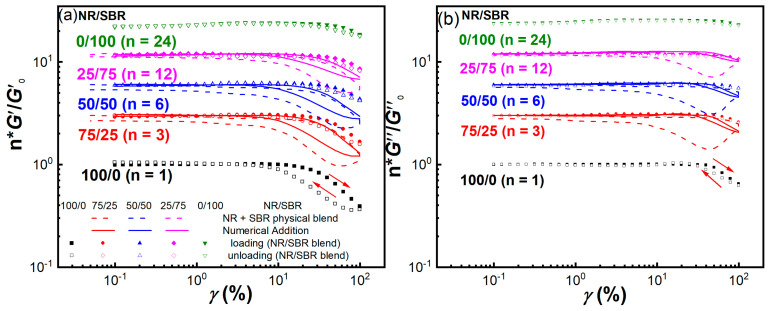
(**a**) *G*′/*G*′_0_ and (**b**) *G*″/*G*″_0_ as a function of *γ* for NR/SBR blends (symbols) and NR + SBR physical blends (dashed lines) during a loading–unloading cycle at 100 °C and 1 rad/s. The solid lines are predicted by numerical addition results of neat NR and SBR. Here, N is also the vertically shifting factor for the normalized moduli. The arrows indicate the loading and unloading processes.

To further explore the time-dependent behavior of the Payne effect for NR/SBR blends with a heterogeneous structure, cyclic strain amplitude sweeps are applied to the NR/SBR blends and NR + SBR physical blends during a loading–unloading cycle at 100 °C and *ω* = 1 rad/s. Figure 7 shows the *γ* dependence of normalized moduli *G*′/*G*′_0_ and *G*″/*G*″_0_ undergoing cyclic oscillation deformation. In the investigated *γ* region, neat NR exhibits more remarkable *G*′ and *G*″ recovery hysteresis than neat SBR during a cyclic sweep at *γ* > 10%. To compare the difference of modulus recovery hysteresis for NR/SBR blends and NR + SBR physical blends, the normalized moduli for the blends with different weight ratios are also vertically shifted by a factor of n. With the increase in SBR content, the *G*′ hysteresis of the NR/SBR blends decreases significantly, and it is much smaller than those for the corresponding NR + SBR physical blend and the numerical addition of neat NR and SBR. Furthermore, the *G*″ hysteresis of the NR/SBR blends almost disappears, while that of the NR + SBR physical blends is even more marked than that of neat NR. Hence, the heterogeneous gel-like structure in the blends may suppress the mechanical hysteresis upon large-*γ* cyclic sweeps, especially in the co-continuous NR/SBR (50/50) blend.

**Figure 8:**
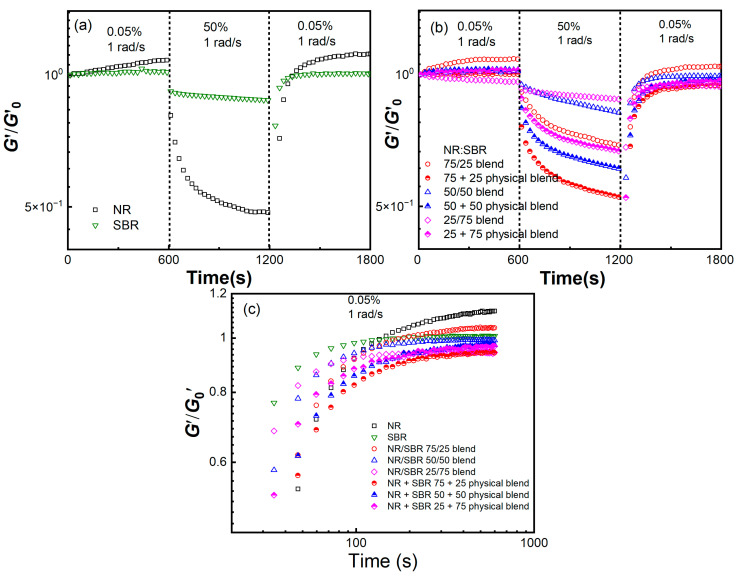
*G*′*/G*′_0_ as a function of lasting time for (**a**) pure NR and SBR, (**b**) NR/SBR blends and NR + SBR physical blends being sheared stepwise to *γ* = 0.05%, 50% and 0.05 at *ω* = 1 rad/s. (**c**) is extracted from (**a**,**b**) for clearly showing the normalized modulus recovery process for NR, SBR, NR/SBR blends and NR + SBR physical blends at *ω* = 1 rad/s from large to small strain steps (*γ* = 50% shear to *γ* = 0.05%).

In the LAOS test, the Payne effect is manifested as a decrease in dynamic modulus with increasing periodic oscillatory strain. The decrease in the modulus corresponds to the change in the microstructure of the system. Once the loaded strain is removed, the material will undergo a time-dependent structural adjustment. Therefore, the time-dependent characteristics of the Payne effect could be explored from another perspective on the modulus recovery process upon loading a large strain. Here, the NR, SBR, and NR/SBR blends, as well as the NR + SBR physical blends, were sheared stepwise to *γ* = 0.05% and 50%, successively, and then sheared back to *γ* = 0.05% at 100 °C and *ω* = 1 rad/s, with each loading step lasting for 600 s. Figure 8 shows the normalized *G*′ as a function of lasting time. It could be seen that when *γ* = 0.05%, the *G*′*/G*′_0_ of the SBR and NR + SBR physical blends and the NR/SBR (50/50), (25/75) blends keep almost constant, and that the *G*′*/G*′_0_ of the NR and NR/SBR (75/25) blend increases slightly, where *G*′_0_ is the initial modulus. When the strain is switched to 50%, the *G*′*/G*′_0_ of all the samples decays rapidly, corresponding to the transition from the solid-like to liquid-like characteristics [46]. The attenuation amplitude of NR/SBR blends decreases with increasing SBR content, and is weaker than that of NR + SBR physical blends, meaning that it is consistent with the loading–unloading experimental results shown in Figure 6 and Figure 7. After being sheared back to *γ* = 0.05%, all the samples exhibit a gradual modulus recovery, while the NR/SBR blends show a more rapid recovery process than the NR + SBR physical blends (as shown in Figure 8c), revealing that the heterogeneous structure in NR/SBR blends may improve the reversibility of the Payne effect.

In the LAOS test, the *G*′_0_ and *G*″_0_ of NR are obviously higher than those of SBR, and the *γ*_c_ of NR is much smaller than that of SBR. Hence, the heterogeneous structure of NR/SBR blends may result in the heterogeneity of microscopic deformation for the NR and SBR domains. Such a phenomenon is similar with the strain amplified effect of filled rubber [47]. However, the modulus of rigid particles is far greater in that of the filled rubber and the particles are not deformed, resulting in the occurrence of stain amplification in matrix rubber. Here, the modulus difference between NR and SBR is obviously smaller than that between rigid particles and rubber. Namely, the microscopic deformation of SBR domain is larger than the macroscopic deformation of the NR/SBR blends, while the microscopic deformation of the NR domain is smaller. Hence, the modulus decay and hysteresis of the NR/SBR blend during loading is remarkably smaller than that of the numerical addition and the NR + SBR physical blend. Furthermore, the domain deformation from the equilibrium morphology of a gel-like structure to an elongated morphology under large *γ* may result in the increase in interfacial energy and then the additional contribution of storage term [48,49]. Hence, the co-continuous NR/SBR (50/50) blend with large interfacial area has the smaller modulus decay and hysteresis during the loading–unloading process.

### 2.4. Fourier-Transform Rheology Analysis

**Figure 9:**
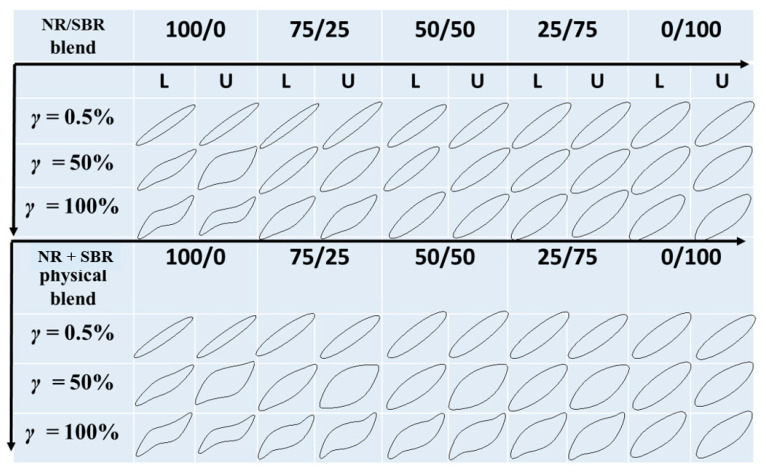
Normalized Lissajous curves generated from oscillatory shearing measurements at different strain amplitudes for NR/SBR blends and NR + SBR physical blends at 100 °C and 1 rad/s. Here, L represents loading and U represents unloading.

Lissajous curves are a way to transform the time domain of stress into a stress–strain domain, and can be also used to reflect the nonlinear characteristics of the materials under LAOS on the basis of closed loops for normalized stress versus normalized strain [50]. Figure 9 shows the Lissajous curves of the NR, SBR, and NR/SBR blends, as well as the NR + SBR physical blends, at 100 °C and *ω* = 1 rad/s. The Lissajous curves of all the samples at low strain amplitude are elliptical, corresponding to the predominately elastic stress response. As the strain increases, the Lissajous curves deviate significantly from the ellipse due to strain softening [51]. The distortion at mediate and high *γ*s for NR/SBR blends is dependent on the SBR content and the blend morphology, while the distortion for the NR + SBR physical blends is very close to that of pure NR. The distortion of SBR is much weaker than that of NR, and the presence of SBR in the NBR/SBR blend may weaken its distortion. It should be noted that the Lissajous curves for the co-continuous NR/SBR (50/50) blend are even able to almost maintain an elliptic shape at high *γ* (100%), indicating that the microscopic strain amplification of SBR and the additional elastic contribution which resulted from the co-continuous domain deformation may weaken their strain softening. During the unloading process, it could be observed that the NR + SBR physical blends also show more obvious elliptical distortion than the NR/SBR blends with the corresponding composition, indicating that the recovery of NR + SBR physical blends is remarkably slower than that of the NR/SBR blend, and that the microscopic heterogeneous structure in NR/SBR blends may weaken their nonlinear viscoelastic and elliptical distortion characteristics.

**Figure 10:**
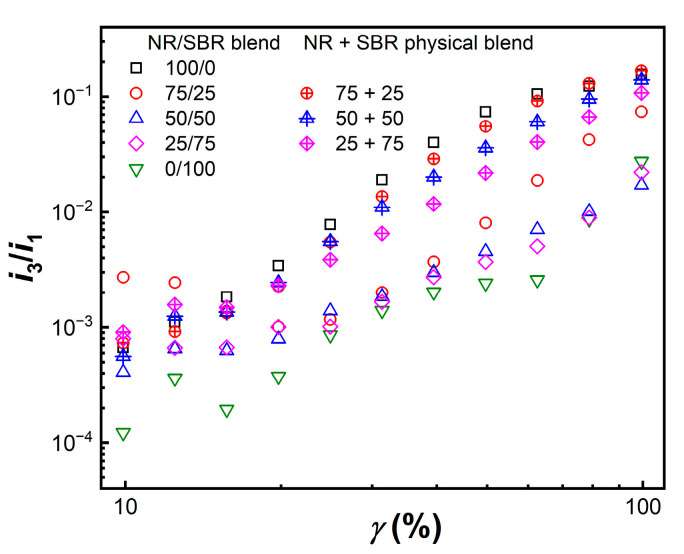
Relative third harmonic (*i*_3_/*i*_1_) as function of strain amplitude (*γ*) for NR/SBR blends and NR + SBR physical blends at 100 °C and 1 rad/s.

In order to further explore the effect of blend morphology on their nonlinear viscoelastic response, the high-order harmonics *i*_n_ can be separated by using a Fourier transformation of the stress and strain signals. The stress (*σ*(*t*)) could be expressed as follows [52]:

Σ*i*_n_sin(*nω*_0_
*t* + *δ*_n_) (2)

where *ω*_0_ is the frequency of the fundamental wave, and δ_n_ is the phase angle. Among the high-order harmonics, the intensity of odd-numbered harmonics is higher than that of even-numbered harmonics, and the intensity of harmonics gradually decreases as the order increases. Since the third harmonic contributes the most to the nonlinearity, the ratio of the intensity of the third harmonic (*i*_3_) to the intensity of the first harmonic (*i*_1_) (*i*_3_/*i*_1_) is generally used to quantify the degree of nonlinearity. Figure 10 shows the *γ* dependence of *i*_3_/*i*_1_ for the NR/SBR blends and the NR + SBR physical blends. It could be seen that the two blend systems exhibit slightly different nonlinear characteristics, and that the nonlinearity of NR is more obvious than that of SBR. Furthermore, the *i*_3_/*i*_1_ of the two systems generally increase with the increase in *γ*, which is attributed to the orientation and stretch of polymer chains [53]. Here, *i*_3_/*i*_1_ for the NR + SBR physical blends is close to that of virgin NR, while *i*_3_/*i*_1_ for NR/SBR blends is similar with that of virgin SBR, indicating that the microscopic heterogeneous structure in NR/SBR blends may weaken their nonlinearity [54].

## 3. Conclusion

The effect of blend morphology on the linear and nonlinear rheological response for NR/SBR blends and NR + SBR physical blends is investigated. The increment of linear viscoelastic response for NR/SBR blends at low *ω*s in the nonterminal region is mainly related to their heterogeneous structure. The Payne effect and the modulus hysteresis of NR + SBR physical blends are close to those of virgin NR, and much more obvious than those of the NR/SBR blends. The heterogeneous gel-like structure in NR/SBR blends may result in the heterogeneity of microscopic deformation for the NR and SBR phases, and the additional elastic contribution which resulted from the increasing interfacial energy of domain deformation, which may further weaken their strain softening. This is especially true for the NR/SBR (50/50) blend, where the microscopic strain amplification of the SBR phase and the additional elastic contribution resulted from the co-continuous domain deformation may jointly lead to the weak nonlinear viscoelastic response and fast modulus hysteretic recovery under a large *γ*. These results may provide some new evidence for illustrating the Payne effect mechanism for rubbery blends.

## 4. Experimental

### 4.1. Materials

Natural rubber (NR, Vietnam SVR-3L standard rubber, *M*_w_ = 1.12 × 10^6^, *M*_w_/*M*_n_ = 3.57) was purchased from Shanghai Duokang Industry. Co., Ltd., Shanghai, China. Styrene butadiene rubber (SBR1739, *M*_w_ = 6.4 × 10^5^, *M*_w_/*M*_n_ = 2.7, styrene content = 40%, vinyl content = 18%) was purchased from Shenhua Chemical Industry Co., Ltd., China. Rubber antioxidant *N*-1,3-dimethylbutyl-*N’*-phenyl-*p*-phenylenediamine (6PPD) was purchased from Shanghai Macklin Biochemical Technology Co., Ltd., Shanghai, China.

### 4.2. Sample Preparation

The NR/SBR blends were prepared by using a laboratory internal mixer (Siemens Electric Co., Ltd., Beijing, China) at 110 °C for 10 min. A total charge of rubber was 50 g, and the rotor speeds was set as 60 rpm. The mass ratios of NR and SBR were 100/0, 75/25, 50/50, 25/75, and 0/100, respectively. Antioxidant (6PPD) of 1.5% by weight of total rubber was added in the early stages of mixing. The blends were compressed into sheets of 2 mm in thickness and a diameter of 25 mm in a metal mold under 14.5 MPa on a press vulcanizer (XL-25, Huzhou Xinli Rubber Machinery Co., Ltd., Huzhou, China) at 110 °C for 20 min.

In order to explore the contribution of the domain interface for NR/SBR blends to their viscoelastic response, we prepared the control samples by direct hot-pressing in proportion without internal mixing. The schematic illustrations of sample combination modes were given in Figure 1. First, the thin sheets obtained by the hot-pressing of pure NR and SBR were cut into 25 mm diameter discs with a cutter, and then the NR and SBR discs with a thickness of 2 mm and a diameter of 25 mm were cut and combined by lateral splicing according to the weight ratios, respectively. The spliced rubber sheets according to the weight ratio were put in a metal mold, and then compressed at 110 °C for 20 min under a press vulcanizer (XL-25, Huzhou Xinli Rubber Machinery Co., Ltd., Huzhou, China) with a pressure of 14.5 MPa. The thickness and diameter of the NR + SBR physical blends rubber sheets were 2 mm and 25 mm, respectively. Owing to the different moduli and viscosity of NR and SBR, their different weight ratios might also affect their rheological response.

### 4.3. Characterizations

Dynamic mechanical analysis (DMA, Q800, TA Instruments, New Castle, DE, USA) was also used to measure the glass transition temperature of the NR, SBR, and NR/SBR blends in the dual cantilever mode. The length, width, and thickness of the samples were 35 mm, 13 mm, and 2 mm, respectively. The dynamic strain amplitude, frequency, and heating rate employed were 50 μm, 10 Hz, and 3 °C/min, respectively.

The morphology of the NR/SBR blends was observed using a transmission electron microscope (TEM, JEM-1230, JEOL Company, Japan) at an acceleration voltage of 120 kV, after the blends were made into samples about 90 nm in thickness by freezing and ultrathin slicing.

The linear and nonlinear dynamic rheological responses of samples at 100 °C were measured using a strain-controlled rheometer (ARES-G2, TA Instruments, New Castle, DE, USA). The stainless-steel clamp was equipped with a serrated surface texture to prevent rubber slippage. Dynamic frequency (*ω*) sweeps were performed from 100 to 0.01 rad/s in the linear viscoelastic regime (0.5% strain amplitude). Dynamic strain amplitude (*γ*) sweeps were performed from 0.05% to 100% and then from 100% to 0.05% at *ω* = 1 rad/s to investigate the nonlinear viscoelasticity [55,56]. The time dependence of rheological recovery was obtained by shearing NR, SBR, and their blends stepwise to *γ* = 0.05%, 50% and 0.05% at *ω* = 1 rad/s.

## Data Availability

Data available on request from the authors.
